# The Effect of Socioeconomic Disparities on Prefrontal Activation in Initiating Joint Attention: A Functional Near-Infrared Spectroscopy Evidence From Two Socioeconomic Status Groups

**DOI:** 10.3389/fnhum.2021.741872

**Published:** 2021-12-10

**Authors:** Keya Ding, Chuanjiang Li, Yanwei Li, Hongan Wang, Dongchuan Yu

**Affiliations:** ^1^Key Laboratory of Child Development and Learning Science of Ministry of Education, School of Biological Science and Medical Engineering, Southeast University, Nanjing, China; ^2^Hangzhou College of Early Childhood Teacher’s Education, Zhejiang Normal University, Hangzhou, China; ^3^College of Preschool Education, Nanjing Xiaozhuang University, Nanjing, China; ^4^Department of Child Development and Behavior, The Third Affiliated Hospital of Zhengzhou University, Zhengzhou, China

**Keywords:** socioeconomic disparities, home reading environment, young children, functional near-infrared spectroscopy (fNIRS), initiating joint attention

## Abstract

Low socioeconomic status (SES) may generally have a long-lasting negative effect on cognitive development, and show deficits in the development of executive functions. However, it is unclear whether there is an SES-dependent disparity in the functional brain development of the prefrontal cortex. By collecting task-related functional near-infrared spectroscopy (fNIRS) data and behavioral data (e.g., intelligence, language, home reading environment (HRE), family income, and parental education level), the current study aimed to detect whether the SES of preschool children (*N* = 86) is associated with prefrontal activation during the joint attention task. Results verified that low-SES children show lower right prefrontal activation during joint attention than Relatively High-SES children. In addition, our findings confirmed the mediating effect of HRE on the association between SES and brain activation during joint attention, as well as that between SES and language ability. These results suggest that SES contributes to functional development of the prefrontal regions, and the improvement of HRE could be a potential strategy to intervene SES-related disparities on child development.

## Introduction

Socioeconomic status (SES) has long been recognized as an important determinant of cognitive and academic functioning throughout life. Children from low-SES backgrounds generally have a poor executive function and thereby, lead to low psychometric intelligence, working memory capacity, reading skills, literacy, and academic performance. By using brain imaging tools such as magnetic resonance imaging (MRI) and electroencephalography (EEG), some scientists have just investigated the effect of SES-dependent disparity on brain development recently (Moriguchi and Shinohara, 2019). Available evidence (Moriguchi and Shinohara, 2019; Judd et al., 2020; Ramphal et al., 2020; Tooley et al., 2021) support that low-SES may contribute to unexpected abnormal changes in brain structure, functions, connectivity, and activation pattern, particularly in the prefrontal cortex (PFC). For instance, MRI research revealed that compared with normal children, low-SES children have lower regional gray matter volume (Hair et al., 2015). In addition, a resting-state EEG study showed that children from low-income homes exhibit lower alpha power and higher theta power than their counterparts from relatively higher-income homes (Maguire and Schneider, 2019). Remarkably, previous studies focused on the resting-state brain structure and functions (task-free). The current study aimed to analyze task-related brain imaging data and bring new insights into the understanding of brain structural and functional changes underlying childhood SES disparities.

Childhood SES is the social standing or class of an individual or group. Measurement of childhood SES is complex, and the most common indicators are family income, parental education, and parental occupation (McLoyd, 1998). Some scientists have just investigated the association between SES measures and brain development recently (Judd et al., 2020; Noble and Giebler, 2020; Tomasi and Volkow, 2021). Typical results include that: (i) Children from low income families may have a lower frontal, temporal, and hippocampal volume, as well as smaller brain surface area; (ii) Children from family with low parental education may have a lower cortical thickness in frontal areas; and (iii) Higher parental education level may be associated with significantly increased volume in the fetal white matter, deep gray matter, and brainstem. It should be remarked that early physical and psychosocial environment (generating stimuli), associated with SES, may play a significant role in cognitive and social development. As one of the important physical and psychosocial environments, home reading environment (HRE), consisting of the literacy resources and adult-child interactions, has been verified its significant influence on children’s brain development, language, and social cognition (Chow et al., 2017; Dolean et al., 2019). Indeed, researches showed that children with better HRE may evoke higher activation in the left parietal-temporal-occipital association cortex (Hutton et al., 2015). However, the relationship among HRS, SES, cognitive development (especially language development), and brain development remains unclear.

Initiating joint attention (IJA) defines as the ability of an individual to spontaneously seek to direct the attention of a social partner, and to share their experience of an object or event. The IJA involves aspects of executive attention regulation, inhibitory control, and self-monitoring that are critical for the subsequent development of cognitive, social, and learning skills (Nummenmaa and Calder, 2009; Redcay et al., 2012). The IJA behavior may evoke widespread activation in brain regions related to attention, social cognition, decision-making, emotion, and motivation (Redcay et al., 2010 and Caruana et al., 2015), such as PFC, middle frontal gyrus (MFG), inferior frontal gyrus (IFG), temporoparietal junction (TPJ), and precuneus. Remarkably, previous IJA-related imaging results focused on infants and adults. The researches on IJA-related imaging data are not sufficient for preschool children, particularly for low-SES children.

Taken together, this study aimed to detect the effect of SES disparities on prefrontal activation during IJA tasks for two kinds of preschool children (i.e., low-SES children from village and Relatively High-SES children from suburban), by the functional near-infrared spectroscopy (fNIRS). The reason for using fNIRS is due to its safety, portability, relatively high temporal resolution, insensitivity to head movement, and reliable clinical utility. As the main motivation, the current study intended to detect two hypotheses: (i) SES of preschool children is associated with prefrontal activation during the IJA task; and (ii) HRE mediates the influence of SES on brain activation and cognitive abilities (e.g., language). This study also discussed the group differences of behavioral measures (associated with SES, HRE, intelligence, and language).

## Materials and Methods

### Procedure

The participants were recruited from a kindergarten in a village (corresponding to the Low-SES group) and from a kindergarten in a suburban area (corresponding to the Relatively High SES group) in China, respectively. The interested families contacted the teachers to confirm their understanding of the research content and completed a brief screening to determine eligibility. Children were excluded from participants in this study if they had a history of a neurological disorder, loss of consciousness, sensory impairments, autism spectrum disorder, or an intellectual disability. All study procedures and research methods were carried out in accordance with the Declaration of Helsinki (Rickham, 1964) by the World Medical Association concerning human experimentation, and were approved by the Research Ethics Committee at Southeast University. Written informed consent was obtained from the caregivers, and oral consent was obtained from the children before starting the task. Each participating child received an age-appropriate toy after experiments.

### Participants

Although 104 preschool children were recruited in the study, 10 subjects were excluded due to loss of fNIRS data, unacceptable head movement, or refusal to attend the experiments. Eight children were further excluded because their parents did not complete all questionnaires as expected. Therefore, only 86 participants (45 males) aged 40–75 months (60.9 ± 7.61 months) were actually considered in this study.

### Socioeconomic Status-Related Information Collection

For each participated child’s parents, they were asked to complete two questionnaires, i.e., the Family Basic Information Questionnaire (FBIQ) and the Home Reading Environment Parental Questionnaire (HRE-PQ), to collect the basic information of participants, SES and HRE.

Parental education level was assigned a value from 1 to 7 as follows: 1, less than primary school; 2, middle school; 3, high school; 4, technical secondary school; 5, college; 6, undergraduate degree; and 7, graduate level or above. In addition, the monthly family income (in Chinese RMB) was assigned a value from 1 to 7 by the following rule: 1, 2,000 or below; 2, 2,001–4,000; 3, 4,001–6,000; 4, 6,001–8,000; 5, 8,001–12,000; 6, 12,001–15,000; and 7, >15,000.

The number of storybooks in the home, the frequency of parent-child reading, and days of reading per week were considered as factors of the HRE measure. The number of storybooks in home was assigned a value from 1 to 9 as follows: 1, ≤10; 2, 11–20; 3, 21–50; 4, 51–80; 5, 81–110; 6, 111–140; 7, 141–170; 8, 171–200; and 9, >200. While, the frequency of parent-child reading at home was coded from 1 to 5 (1 = never, 2 = rarely/occasionally, 3 = sometimes, 4 = often, 5 = very often).

### Cognitive Measures

For participated children, they were asked to complete an intelligence test and a Chinese receptive language task by the Combined Raven’s Test (CRT) (Ming, 1989) and Peabody Picture Vocabulary Test-Revised (PPVT) (Lu and Liu, 1998), respectively.

### The Initiating Joint Attention Task

**FIGURE 1:**
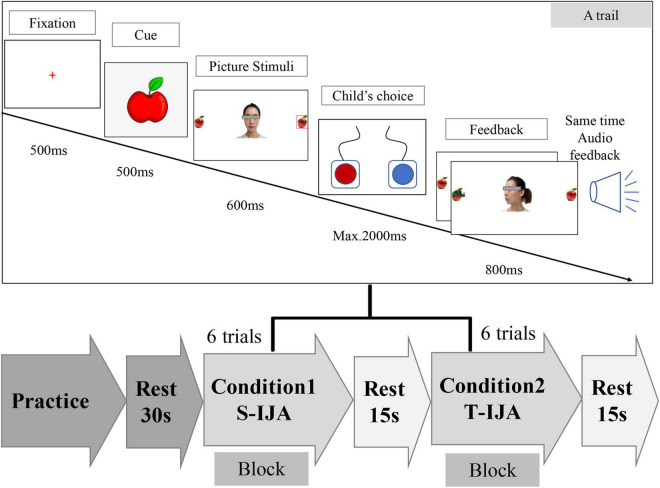
The Initiating Joint Attention (IJA) task schematic. The experiment consisted of two kinds of social partners (i.e., stranger and teacher) in the IJA task, and considered two conditions, i.e., Condition 1 (S-IJA, corresponding to interaction with the stranger), and Condition 2 (T-IJA, corresponding to interaction with the teacher). Each condition was a block that included 6 trials and would be repeated three times in the experiment. In an IJA trial (see the top of the figure), participants were asked to freely choose to shift the gaze of the interacting partner (teacher/stranger) by pressing the left or right button. The partner (teacher/stranger) shifted his/her gaze leftward or rightward dependent on the participant’s choice.

The experimental protocol (including 2 conditions) was clearly shown in Figure 1, where each condition included 3 repeated blocks and each block contained 6 trials. A game-like and child-friendly paradigm was used to explore children’s brain activity during the IJA task under two conditions, i.e., T-IJA (corresponding to teacher-child interactional case) and S-IJA (corresponding to the stranger-child interactional case). The photographs from both participant’s close teacher and a female stranger were utilized to imitate the social interaction with familiar or unfamiliar partners, respectively. These photographs were gathered according to the following rules. For each teacher or stranger, five photographs were taken, for which three of them were with the gaze turned forward, leftward, rightward, while two of them were happy and sad faces used as feedback to correct or incorrect responses.

As shown in Figure 1, each block (including 6 trials) was repeated three times in the experiment. For a trail, the cue (an apple picture) was first present in the middle of the computer screen for 500 ms. Then, the photography of the interactive partner (teacher/stranger) looking toward the participant was present in the midline of the computer screen, with two apples located on the left and right sides. The participant was asked to freely choose to shift the interacting partner’s gaze leftward or rightward by pressing the left or right button (maximum of 2,000 ms), respectively. After that, the participant received verbal feedback “Yeah!”, indicating that the interacting partner followed the participant’s gazing.

The IJA task with a block design was programmed and conducted with the software E-prime (Psychology Software Tools Inc., Pittsburgh, PA, United States, Version 1.0). The whole experiment (lasting 7–8 min) was presented on a 14-inch computer screen (resolution 1,024 × 768) with a white background. Before a formal experiment, participants were instructed to do some practice for the understanding of the whole experimental procedure under the standard exceeding 80% accuracy.

### Functional Near-Infrared Spectroscopy Data Acquisition

**FIGURE 2:**
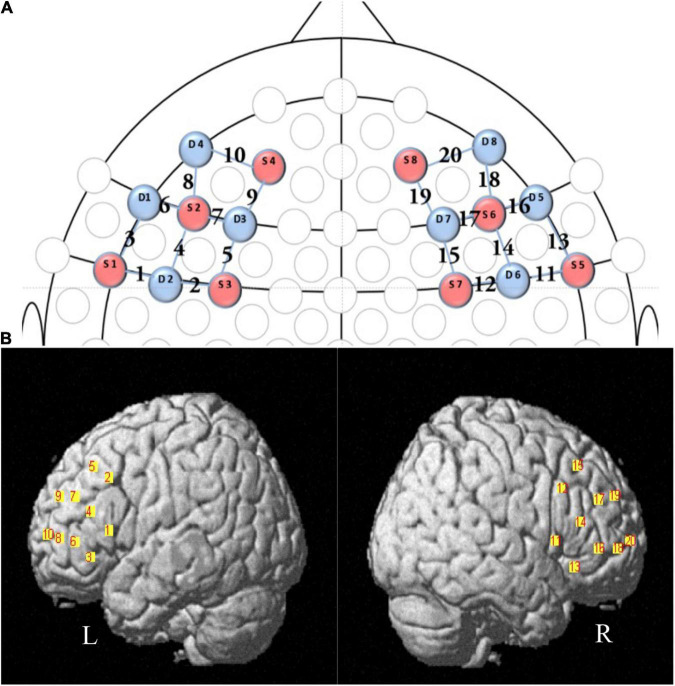
fNIRS channel and probe configuration. **(A)** The 2D map illustrated the distribution of probes (red and blue representing the source and detector probe, respectively) and 20 channels. **(B)** The 3D map showed the positioning of channels. L, the left hemisphere of brain; R, the right hemisphere of brain; IFG, inferior frontal gyrus; MFG, middle frontal gyrus.

During IJA tasks, the oxy-hemoglobin (HbO) and deoxy-hemoglobin (HbR) concentration changes at the wavelengths of 630 and 850 mm were recorded using an fNIRS equipment, i.e., NIRSport 8*8 (NIRx Medical Technology LLC, Glen Head, NY, United States) Optical Topography System, with sampling frequency 7.81 Hz. This fNIRS equipment had 8 light sources and 8 detectors, evenly distributed over the left and right prefrontal regions according to the 10-10 transcranial positioning system. Hence, this fNIRS equipment had 20 neural channels covering the bilateral inferior frontal gyrus (IFG) and middle frontal gyrus (MFG) (see Table 1 and Figure 2 for detailed information).

**TABLE 1:** Channels corresponding to BA/AAL/MNI label.

Channel	BA	AAL	MNI (x, y, z)
CH1	44, 45	Triangular IFG	−60, 19, 11
CH2	47	IFG	−55, 32, −5
CH3	9, 10	MFG	−38, 52, 29
CH4	10	MFG	−39, 61, 7
CH5	8, 9	MFG	−43, 29, 44
CH6	9	MFG	−53, 21, 38
CH7	46	MFG	−46, 43, 28
CH8	10, 46	MFG	−46, 53, 6
CH9	45, 46	Triangular IFG	−55, 31, 20
CH10	47	Triangular IFG	−53, 42, 3
CH11	47	Triangular IFG	56, 41, 1
CH12	10, 46	MFG	49, 53, 2
CH13	45, 46	Triangular IFG	60, 30, 16
CH14	46	MFG	49, 43, 28
CH15	10	MFG	42, 62, 5
CH16	9, 10	MFG	40, 52, 30
CH17	9	MFG	58, 19, 34
CH18	8, 9	MFG	46, 28, 46
CH19	47	IFG	55, 27, −8
CH20	44, 45	Opercular IFG	63, 13, 8

*CH, channel; BA, Brodmann area; AAL, automated anatomical label; IFG, inferior frontal gyrus; MFG, middle frontal gyrus.*

### Pre-processing

**FIGURE 3:**
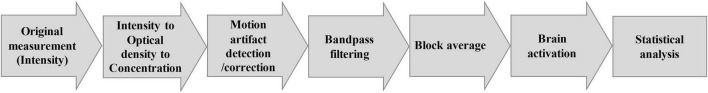
Data processing workflow.

The fNIRS data were preprocessed by the Homer2 toolbox (Huppert et al., 2009) (see Figure 3 for detailed flowchart). As shown in Figure 3, after raw intensity data being converted to optical density units, checking and repairing steps were conducted by the channel artifact detection and spline correction method, in order to ensure data quality even in the presence of large artificial signal variations. Then, high- and low-frequency noise of signals was removed by a bandpass filter with cutoff frequencies at 0.01–0.2 Hz. Finally, hemoglobin concentration variations were calculated by a modified Beer-Lambert law (Delpy et al., 1988).

The classic hemodynamic responses, characterized by an increase in HbO and a corresponding decrease in HbR, were observed and identified. The activation peaks in each trail were also recorded and calculated. This study considered HbO concentration only, due to its high sensitivity to changes in regional cerebral blood flow. In addition, the time window of interest was set at the range of 11–21 s for each condition (block). Therefore, HbO concentration changes at the range of 11–21 s were actually taken into account for time-series analysis.

### Statistical Analysis

Participants were divided into two SES groups, i.e., Low-SES and Relatively High-SES groups, according to their monthly family income and place of residence. The Low-SES group was corresponding to children with monthly family income below 8,000 RMB and living in the rural area; while the Relatively High-SES group was corresponding to children with monthly family income above 8,000 RMB and living in the suburban area.

The experiments in this study included two factors, i.e., partner-types (teacher and stranger) and SES-types (Low-SES and Relatively High-SES). By SPSS (IBM Corporation, Armonk, NY, United States), a two-factor ANOVA (with 2 partner-types*2 SES-types) was conducted to examine the main effects for prefrontal activation, with the significance level 0.05. The false discovery rate (FDR) correction was conducted for results with multiple comparisons (Benjamini and Hochberg, 1995).

Comprehensive evaluations of SES and HRE levels were conducted by the factor analysis. In particular, it was required to calculate the regressed function (i.e., a weighted linear operator) of SES level on three factors (i.e., mother’s education level, father’s education level, and family monthly income), as well as the regressed function (i.e., a weighted linear operator) of HRE level on three factors (i.e., the number of storybooks in home, the frequency of parent-child reading at home, and days of reading per week).

The mediating effect of HRE on the association relation between SES and brain activation, as well as that between SES and cognitive abilities (e.g., language), were also detected. The correlations between behavioral measures (i.e. SES, HRE, intelligence, and language) and prefrontal activation were analyzed as well.

## Results

### Behavioral Analysis

Table 2 showed the differences in behavioral measures between Low-SES and Relatively High-SES groups. It is easy to see from Table 2 that there was no significant age difference between the two groups (*t* = 1.993, *p* = 0.05). In addition, for each behavioral measure, Relatively High-SES children had a significantly higher score than Low-SES children (*p* < 0.05).

**TABLE 2:** Demographic characteristics of participants.

	Low-SES children	RH-SES children
Age (months)	62.40 ± 6.2	59.08 ± 8.76
Monthly family income	2.79 ± 1.25	5.69 ± 0.66
Mother’s education level	2.07 ± 0.88	4.92 ± 1.278
Father’s education level	2.32 ± 0.77	4.95 ± 1.45
Number of storybooks in the home	2.2 ± 1.27	6.97 ± 1.98
Days of reading per week	1.32 ± 1.49	4.14 ± 1.46
Frequency of parent-child reading at home	2.14 ± 1.13	4.22 ± 1.08
CRT score	16.49 ± 4.05	21.35 ± 8.003
PPVT score	43.13 ± 14.73	57.83 ± 25.89

*SES, socioeconomic status; RH-SES, Relatively High-SES; CRT, Combined Raven’s Test; PPVT, Peabody Picture Vocabulary Test-Revised.*

By the factor analysis, we verified that: (i) the SES level may be predicted by the weighted sum of family income (with weight coefficient β = 0.87), mother’s education level (with weight coefficient β = 0.95), and father’s education level (with weight coefficient β = 0.93); and (ii) the HRE variable may be predicted by the weighted sum of the number of storybooks in the home (with weight coefficient β = 0.93), days of reading per week (with weight coefficient β = 0.93), and the frequency of parent-child reading at home (with weight coefficient β = 0.94).

We interestingly found (see Table 3) that there was a significantly high correlation between HRE and SES (*r* = 0.843, *p* < 0.001). Additionally, the PPVT score was correlated with SES level (*r* = 0.259, *p* = 0.023) and HRE level (*r* = 0.4, *p* < 0.001); while the Raven test score was correlated with SES level (*r* = 0.344, *p* = 0.003) and HRE level (*r* = 0.380, *p* = 0.001).

**TABLE 3:** Correlations between behavioral measures.

Correlation	PPVT score	CPT score	SES	HRE	FI	MEL	FEL	NS	DRW
CPT score	0.460**								
SES	0.259*	0.344**							
HRE	0.400**	0.380**	0.843**						
FI	0.272*	0.291**	0.874**	0.726**					
MEL	0.267*	0.324**	0.948**	0.830**	0.738**				
FEL	0.228*	0.343**	0.928**	0.771**	0.685**	0.861**			
NS	0.379**	0.295**	0.858**	0.926**	0.733**	0.861**	0.772**		
DRW	0.408**	0.432**	0.759**	0.925**	0.686**	0.737**	0.676**	0.773**	
FPCR	0.328**	0.332**	0.734**	0.941**	0.611**	0.715**	0.705**	0.813**	0.812**

PPVT, Peabody Picture Vocabulary Test-Revised; CRT, Combined Raven’s Test; SES, socioeconomic status; HRE, home reading environment; FI, family income; MEL, mother’s education level; FEL, father’s education level; NS, number of storybooks in home; FPCR, frequency of parent-child reading at the home; DRW, days of reading per week. **p < 0.01; *p < 0.05.

### Brain Activation Difference

**FIGURE 4:**
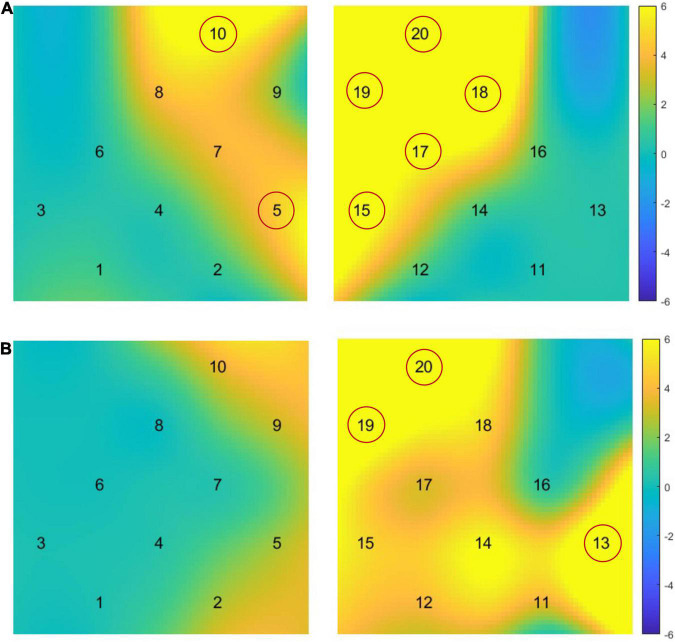
Heatmaps of prefrontal activation difference during the Initiating Joint Attention (IJA) task. **(A)** Heatmap of prefrontal activation difference between two social interaction conditions (i.e., interaction with stranger and that with teacher) in the IJA task. **(B)** Heatmap of prefrontal activation differences between Low-SES and Relatively High-SES children. The heatmaps displayed the interpolated *F*-values, where channels with significant activation differences were highlighted in yellow, and channels with non-significant differences were plotted in blue. L, the left hemisphere of brain; R, the right hemisphere of brain; SES, socioeconomic status.

We conducted a 2 × 2 ANOVA between two SES groups (Low-SES and Relatively High-SES children) under two conditions (i.e., S-IJA and T-IJA) to examine the main effects for prefrontal activation during the IJA tasks. Results showed that the main effect of two joint attention conditions is significant. In particular, compared with T-IJA condition, S-IJA condition evoked higher brain activation in CH5 (*F* = 6.03, *p* = 0.045), CH10 (*F* = 8.04, *p* = 0.02), CH15 (*F* = 8.76, *p* = 0.02), CH17 (*F* = 6.77, *p* = 0.037), CH18 (*F* = 11.92, *p* = 0.01), CH19 (*F* = 8.21, *p* = 0.024), and CH20 (*F* = 24.31, *p* < 0.001), covering bilateral MFG and right IFG (see Figure 4A). Furthermore, the between-group ANOVA analysis verified that compared with Low-SES children, Relatively High-SES children evoked significantly higher brain activation in CH13 (*F* = 15.78, *p* < 0.01), CH19 (*F* = 9.81, *p* = 0.02), and CH20 (*F* = 8.48, *p* = 0.03), covering right IFG (Figure 4B). However, there was no significant interactive effect between two SES groups and two conditions.

### Correlation Between Behavioral Measures and Brain Activation

We conducted the correlation between behavioral measures and brain activation during IJA tasks. We found that SES level was significantly correlated with brain activation in CH14 (*r* = 0.263, *p* = 0.02), CH15 (*r* = 0.278, *p* = 0.013), CH18 (*r* = 0.246, *p* = 0.032), CH19 (*r* = 0.34, *p* = 0.002), and CH20 (*r* = 0.254, *p* = 0.024) in the S-IJA condition, as well as that in CH12 (*r* = 0.227, *p* = 0.044), CH19 (*r* = 0.246, *p* = 0.03), and CH20 (*r* = 0.228, *p* = 0.043) in the T-IJA condition. We also revealed that HRE level was significantly correlated with brain activation in CH14 (*r* = 0.238, *p* = 0.035), CH15 (*r* = 0.293, *p* = 0.009), CH18 (*r* = 0.294, *p* = 0.01), CH19 (*r* = 0.411, *p* < 0.01), and CH20 (*r* = 0.285, *p* = 0.01) in the S-IJA condition, as well as that in CH12 (*r* = 0.280, *p* = 0.012), CH14 (*r* = 0.230, *p* = 0.042), and CH20 (*r* = 0.239, *p* = 0.032) in the T-IJA condition.

Interestingly, we found that the language ability (PPVT score) was significantly correlated with brain activation in CH20 (*r* = 0.296, *p* = 0.007) in the S-IJA condition. In addition, the IQ score (Raven score) was significantly correlated with brain activation in CH15 (*r* = 0.222, *p* = 0.049) in the S-IJA condition, and CH20 (*r* = 0.222, *p* = 0.046) in the T-IJA condition.

### The Mediating Role of Home Reading Environment

**FIGURE 5:**
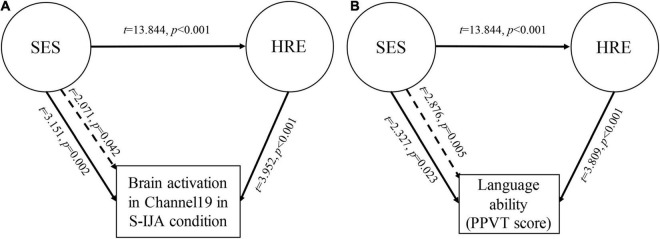
Schematic of mediation models. **(A)** The mediating effect of HRE on the association between SES and brain activation in Channel 19 during initiating joint attention. **(B)** The mediating effect of HRE on the association between SES and language (PPVT score). SES, socioeconomic status; HRE, home reading environment; PPVT, Peabody Picture Vocabulary Test-Revised.

We first evaluated the correlations among SES, HRE, brain activation, and language ability (PPVT score), and then conducted a multiple linear regression to analyze the mediating effect of HRE. Our results confirmed that there is a significant mediating effect of HRE on the association between SES level and brain activation in CH19 (*t* = 2.071, *p* = 0.042) in the S-IJA condition (see Figure 5A). We also found (see Figure 5B) that there is a significant mediating effect of HRE on the association between SES and language ability (PPVT score) (*t* = 2.876, *p* = 0.005).

## Discussion

By collecting task-related fNIRS imaging data as well as behavioral measures (e.g., intelligence, language, family income, parental education level, and home reading environment), the current study aimed to bring some new insights into the understanding of brain structural and functional differences underlying childhood SES disparities. In particular, it was expected to verify whether SES of preschool children is associated with prefrontal activation during joint attention tasks. In addition, this study discussed the mediating effect of HRE on the association between SES and brain activation, as well as the relation between SES and language. To cope with questions above, preschool children with two different SES levels (i.e., Low-SES and Relatively High-SES) were recruited, and IJA tasks were chosen as stimuli to capture fNIRS imaging data associated with social cognition processing.

Results verified that compared with Low-SES children, Relatively High-SES children evoked significantly higher brain activation during joint attention in right IFG. This finding is consistent with previous results that: (i) Low SES contributed to reduced prefrontal activation and affects the functional development of the prefrontal regions (Moriguchi and Shinohara, 2019); (ii) Low-SES contributed to reduced prefrontal-dependent cognitive functions (Kishiyama et al., 2009); (iii) The right IFG played an important role in social cognitive development (Aron et al., 2014); (iv) Childhood SES predicted prefrontal function (i.e., executive function) performance and cortical thickness in the right anterior cingulate (Lawson et al., 2013); (v) Right-prefrontal cortical activity was associated with negative affect, withdrawal motivation, experienced anger and aggression, and emotion regulation (Eddie and Jonathan, 2001; Lee et al., 2012); and (vi) Right-hemispheric prefrontal asymmetry influenced affective recovery and cognitive performance following a failure experience (Haehl et al., 2020). These results suggest that decreased right prefrontal activation during joint attention can be considered as a vulnerability factor for the development of Low-SES children. Based on this inference, appropriate intervention tools will deserve to be investigated in future research.

The current study showed significant brain activation differences between social interactions with a familiar partner (teacher) and that with an unfamiliar partner (stranger) in the IJA tasks. In particular, compared with the familiar interaction case (corresponding to the T-IJA condition), the unfamiliar interaction case (corresponding to the S-IJA condition) evoked higher brain activation in bilateral MFG and right IFG. This result is consistent with previous research that unfamiliar faces evoked significantly higher activation in the right inferior parietal lobule encroaching on the TPJ (Natu and O’Toole, 2011; Matteo et al., 2017). It will deserve to detect in future research if there are some brain regions to support enhanced brain activation in the case of familiar interaction, compared with unfamiliar interaction case.

Relatively High-SES children may usually obtain better home facilities, more opportunities for social interaction with parents, more opportunities for enhancing cognitive abilities, and higher affordability for supporting diverse learning environments (Guryan et al., 2008; Duncan et al., 2011; Brito and Noble, 2014). Our behavioral data confirmed that Relatively High-SES children showed higher intelligence and language level than Low-SES children. This is consistent with previous behavioral studies: (i) Low-SES children had weaker language skills (Raizada et al., 2008); (ii) SES was positively correlated with the language score at the age of 5 years old (Lurie et al., 2021); and (iii) SES was associated with the development of intelligence (Von Stumm and Plomin, 2015). In addition, children from low SES backgrounds may have a long-lasting effect on cognitive development, and show deficits in the development of executive functions (Duncan et al., 1998; Wijeakumar et al., 2019). This remark can be interpreted and supported by our finding that low SES contributes to decreased right prefrontal activation during joint attention.

Longitudinal studies illustrated that better HRE provides more opportunities for teaching and learning activities, and thereby, contributes to better cognitive development (especially language performance in early childhood) (Son and Morrison, 2010; Rodriguez and Tamis-LeMonda, 2011; Niklas and Schneider, 2017; Lurie et al., 2021). We significantly verified the importance of HRE for children’s cognitive development and found that HRE medicates the influence of SES on language and children’s brain activation during IJA tasks. This inference supports that local government should provide more help in enriching HRE and thereby reducing the SES-related disparities in brain development.

Our results showed that: (i) mother’s education level plays the most important role in SES measure and thereby, in cognitive and brain development; and (ii) parental education level is more significant than family income in SES measure. In addition, the factors (i.e., the weighted sum of the number of storybooks in the home, the frequency of parent-child reading at home, and days of reading per week) almost have the same importance in the HRE measure.

Socioeconomic status is a multifaceted structure and its measure is very complex. The most common indicators are family income, parental education, and parental occupation. The SES measure in this study considered only family income and parental education and did not contain the factor of parental occupation, because occupations of low-SES children’s parents are relatively single, basically due to extremely low education level in China. It will deserve in a future study to consider more factors in SES measure, such as food restriction, negative emotionality (e.g., impulsivity and irritability), and motivation (Orsini et al., 2004; Nettle, 2017).

## Conclusion

The current study aimed to investigate the effect of SES on brain activation, and revealed the brain activation difference between Low-SES and Relatively High-SES preschool children during joint attention. Our findings support that low SES contributes to decreased right prefrontal activation during joint attention. In addition, our results verified the significant mediating effect of HRE on the association between SES level and brain activation during joint attention, as well as that between SES level and language ability. Remarkably, a mother’s education level plays the most important role in SES measure.

The current study provides behavioral and neural evidence that SES contributes to cognitive and brain development. The mediating effect of HRE and the role of a mother’s education level in SES measure may bring us some new insights into the understanding of the effect of SES on child development. To reduce SES-related disparities, some potential intervention strategies include the improvement of mothers’ education level, offering libraries, book sharing, e-storybooks, and online storytelling programs for low-SES children.

## Data Availability Statement

The raw data supporting the conclusions of this article will be made available by the authors, without undue reservation.

## Ethics Statement

The studies involving human participants were reviewed and approved by the Institutional Review Board of Southeast University. Written informed consent to participate in this study was provided by the participants’ legal guardian/next of kin.

## Author Contributions

KD, CL, and DY developed the idea for the study. KD and CL collected the data. KD, CL, YL, and HW did the analyses. KD and DY wrote the manuscript. All authors contributed to the article and approved the submitted version.

## Conflict of Interest

The authors declare that the research was conducted in the absence of any commercial or financial relationships that could be construed as a potential conflict of interest.

## Publisher’s Note

All claims expressed in this article are solely those of the authors and do not necessarily represent those of their affiliated organizations, or those of the publisher, the editors and the reviewers. Any product that may be evaluated in this article, or claim that may be made by its manufacturer, is not guaranteed or endorsed by the publisher.
